# Establishment and Thorough Characterization of Xenograft (PDX) Models Derived from Patients with Pancreatic Cancer for Molecular Analyses and Chemosensitivity Testing

**DOI:** 10.3390/cancers15245753

**Published:** 2023-12-08

**Authors:** Diana Behrens, Ulrike Pfohl, Theresia Conrad, Michael Becker, Bernadette Brzezicha, Britta Büttner, Silvia Wagner, Cora Hallas, Rita Lawlor, Vladimir Khazak, Michael Linnebacher, Thomas Wartmann, Iduna Fichtner, Jens Hoffmann, Mathias Dahlmann, Wolfgang Walther

**Affiliations:** 1 Experimental Pharmacology and Oncology GmbH, Robert-Rössle-Str. 10, 13125 Berlin, Germanymathias.dahlmann@epo-berlin.com (M.D.);; 2 CELLphenomics GmbH, Robert-Rössle-Str. 10, 13125 Berlin, Germany; 3 Department of General, Visceral and Transplant Surgery, University Hospital Tübingen, 72076 Tübingen, Germany; 4 Institut für Hämatopathologie, Fangdieckstr. 75, 22547 Hamburg, Germany; 5 ARC-Net Research Center, University and Hospital Trust of Verona, Piazzale A. Scuro 10, 37134 Verona, Italy; 6 NexusPharma, 17 Black Forest Rd., Hamilton, NJ 08691, USA; 7 Clinic of General Surgery, Molecular Oncology and Immunotherapy, University Medical Center Rostock, 18057 Rostock, Germany; 8 University Clinic for General, Visceral, Vascular and Transplantation Surgery, Faculty of Medicine, Otto-von-Guericke-University, 39120 Magdeburg, Germany; 9 Experimental and Clinical Research Center, Charité Universitätsmedizin Berlin, Lindenberger Weg 80, 13125 Berlin, Germany; 10 Max-Delbrück-Center for Molecular Medicine, Robert-Rössle-Str. 10, 13125 Berlin, Germany

**Keywords:** pancreas carcinoma, PDX models, in vivo chemosensitivity testing, molecular profiling

## Abstract

**Simple Summary:**

Patients with pancreatic cancer (PC) still have a poor prognosis as therapeutic options are quite limited for both resectable and inoperable as well as metastasized late-stage tumors. The aim of our study was to generate a PC-PDX in vivo platform of well-characterized models. For this, patient tissues were implanted into immunocompromised mice and established PDXs were examined for morphology, and analyzed regarding their response to standard of care (SoC) drugs, HLA status and molecular characteristics. Our established 45 PC-PDX models closely resemble patient tumor morphology, mutational status, gene expression profile and sensitivity toward gemcitabine, abraxane and 5-FU. Moreover, we show replacement of human tissue by murine stroma, and that stroma components collagen I, α-SMA, FAP and SPARC remain unaffected by the implantation route (s.c. vs. orthotopic) as well as by SoC drug treatment. The comprehensive data set of our PDX models allows testing for new therapeutic targets and novel therapies for PC.

**Abstract:**

Patient-derived xenograft (PDX) tumor models are essential for identifying new biomarkers, signaling pathways and novel targets, to better define key factors of therapy response and resistance mechanisms. Therefore, this study aimed at establishing pancreas carcinoma (PC) PDX models with thorough molecular characterization, and the identification of signatures defining responsiveness toward drug treatment. In total, 45 PC-PDXs were generated from 120 patient tumor specimens and the identity of PDX and corresponding patient tumors was validated. The majority of engrafted PDX models represent ductal adenocarcinomas (PDAC). The PDX growth characteristics were assessed, with great variations in doubling times (4 to 32 days). The mutational analyses revealed an individual mutational profile of the PDXs, predominantly showing alterations in the genes encoding KRAS, TP53, FAT1, KMT2D, MUC4, RNF213, ATR, MUC16, GNAS, RANBP2 and CDKN2A. Sensitivity of PDX toward standard of care (SoC) drugs gemcitabine, 5-fluorouracil, oxaliplatin and abraxane, and combinations thereof, revealed PDX models with sensitivity and resistance toward these treatments. We performed correlation analyses of drug sensitivity of these PDX models and their molecular profile to identify signatures for response and resistance. This study strongly supports the importance and value of PDX models for improvement in therapies of PC.

## 1. Introduction

Pancreatic cancer (PC) is one of the leading causes of cancer deaths and also one of the most lethal malignant neoplasms worldwide [1,2]. PC is associated with a poor prognosis, reflected by the high incidence rate and mortality. With a 5-year survival rate of less than 5% and a median survival of less than 6 months, PC possesses one of the worst prognoses [3,4]. As it develops without well-defined early symptoms, PC is usually diagnosed at advanced stages [5,6]. PC arises either from the exocrine or endocrine parenchyma of the glands. More than 95% of cases occur within the exocrine part and originate from the ductal epithelium, acinar cells or connective tissue. Pancreatic ductal adenocarcinoma (PDAC) is the most common pancreatic neoplasm, which accounts for about 85% of all PC cases. It is an invasive gland-forming, mucin-producing neoplasm with an intensive stromal desmoplastic reaction [7]. The majority of PDACs are formed in the pancreas head with a glandular pattern and different degree of differentiation.

Various risk factors have been identified for the etiology of PC according to epidemiological and genetic studies. PC is an age-associated disease with a mean age at onset of 71 years for men and 75 years for women. Further, PC incidence also differs between males and females, which is 50% higher in men compared to women [8]. More than 80% of PCs develop due to sporadically occurring mutations, while about 10% of cases have a genetic disposition, which increases the individual risk to develop the disease [9]. The increasing risk for PC has been shown to be associated with inherited germline mutations for particular genes, such as Breast cancer 1/2 (BRCA1/2), ataxia–teleangiectasia mutated serine/threonine kinase (ATM), serine/threonine kinase 11 (STK11), serine 1/2 (PRSS1/PRSS2), serine protease inhibitor Kazal-type 1 (SPINK1), the partner and localizer of BRCA2 (PALB2) and DNA mismatch repair genes, such as MutL homolog 1 (MLH1), MutS protein homolog2 (MSH2), MutS homolog 6 (MSH6) and the mismatch repair endonuclease PMS2 [10,11,12,13]. Apart from this, for most familial PCs, the genetic background, however, remains unknown.

Numerous genetic mutations have meanwhile been associated with PDAC, which can be distinguished by the following molecular characteristics: (i) mutational activation of oncogenes, predominantly shown for the V-KI-ras2 Kirsten rat sarcoma viral oncogene homolog (KRAS), being present in more than 90% of PCs; (ii) the inactivation of tumor suppressor genes, such as p53 (TP53) and SMAD family member 4 (SMAD4) present in PanIN-3 lesions or cyclin-dependent kinase inhibitor 2A (CDKN2A); (iii) or inactivation of DNA repair genes, such as MLH1 and MSH2, controlling DNA damage repair and causing microsatellite instability (MSI) due to functional impairment. Further, for PC NRG1 gene fusions, ERBB2 amplifications and BRAF in frame deletions have been identified [14,15,16]. Overall, such genetic alteration represents targets for specific therapies of PC.

Treatment and clinical management of pancreatic cancer are defined by its clinical stage and includes surgery, chemo-radiation therapy, targeted therapy and palliative care [17]. However, current treatment options for PC are insufficient and the outcome for PC has not significantly improved over the past 30 years. Thus, gemcitabine monotherapy still represents the standard of care, which showed a broad range of antitumoral activity [18]. For patients with locally advanced or metastatic PC, gemcitabine has no or little effects on median overall survival [19]. Therefore, many studies aim at gemcitabine treatment in combination with other chemotherapeutic drugs to improve the median survival. While gemcitabine combined with 5-fluorouracil (5-FU), cisplatin or carboplatin did not significantly increase overall survival [20,21,22], a major improvement was demonstrated with FOLFIRINOX combination chemotherapy (oxaliplatin, irinotecan, 5-FU and leucovorin). This combination therapy increased the median overall survival from 6.8 to 11.1 months, compared to gemcitabine alone [23]. In 2013, the American food and drug administration (FDA) approved gemcitabine in combination with nab-paclitaxel for first-line treatment of metastatic PC, which, however, was associated with strong side effects, like peripheral neuropathy or myelosuppression [24,25].

Interfering with the repair of inherent DNA damage by polyADP-ribose polymerase (PARP) inhibitors emerges as a therapeutic concept also for PDAC patients, especially for tumors deficient in homologous recombination [26,27]. In combination with platinum-based therapy, PARP inhibitors already contribute to improved patient survival in metastatic PDAC tumors with inherited BRCA mutations [28,29].

In conclusion, great efforts are still required to significantly improve PC therapy. For this, appropriate and well-characterized models are of the greatest interest. In this regard, patient-derived xenografts (PDXs) represent models closely resembling the clinical appearance and characteristics of human tumors [30,31,32], and numerous PDAC PDX panels of models have been described so far. However, only few PDAC PDX panels have been phenotypically, histologically and molecularly characterized in depth [33,34,35,36]. In fact, such characterization, in association with knowledge on chemosensitivity profiles of PDAC PDX, is the essential basis for the use of these in vivo models to identify novel biomarkers and to screen for therapeutic vulnerabilities of PDAC [37]. In this manuscript, we present data on our stably established PDAC PDX panel, their thorough biological, drug sensitivity and molecular characterization and their potential in drug screening and target/biomarker identification.

## 2. Materials and Methods

### 2.1. Human Pancreas Carcinoma Tissues

An approval of the local ethical committees was given to obtain human pancreas carcinoma specimens, and informed consent was obtained from all patients prior to sample acquisition and experimentation. All patient data were used in an anonymized fashion, according to the ethical guidelines.

### 2.2. Establishment of Human Pancreas Carcinoma Patient-Derived Xenograft (PDX) Models

Fresh surgical tumor fragments of pancreas carcinomas were transplanted subcutaneously (s.c.) into the left flank of anesthetized female NOD scid gamma (NSG) mice. Mice were maintained under sterile and controlled conditions (22 °C, 12 h light–dark cycle, 50% relative humidity, acidified drinking water, autoclaved food and bedding) and observed for a maximum of 3 months. Tumor growth was measured in two dimensions with a digital caliper. Tumor volumes (TVs) were determined with the formula TV = (width^2^ × length) × 0.5. After stable engraftment of PDX, tumors were further passaged in female NMRI nu/nu mice. Tumor PDXs were routinely passaged at TV = 1 cm^3^. The tumor doubling time (TDT) was calculated with GraphPad Prism using the function “exponential growth equation”. The calculations are based on the tumor volume data of 5 untreated mice, each with the same PDX passage number. For vital and sample storage, PDX tumor material was snap-frozen and stored at −80 °C, or processed in addition to formalin-fixed, paraffin-embedded (FFPE) blocks. 

For establishment of orthotopic PDAC, NMRI nu/nu mice were anesthetized, and their peritoneum was laterally incised to exteriorize the pancreas. PDX-derived PDAC tumor fragments were transplanted directly into the pancreas. The pancreas was carefully placed back, the peritoneum was closed with a Surgicryl^®^ (SMI, St.Vith, Belgium) absorbable suture and skin was clamped twice. After approximately 2 weeks, tumors were detectable to monitor tumor growth with ultrasound. Animals were IVC-housed under sterile and standardized conditions (22 °C ± 1 °C, 50% relative humidity, 12 h light–dark cycle, autoclaved food, bedding material and tap water ad libitum). The work conducted in living animals is in accordance with the German Animal Welfare Act as well as the UKCCCR (United Kingdom Coordinating Committee on Cancer Research) and all procedures were approved by local authorities (Landesamt für Gesundheit und Soziales, LaGeSo Berlin, Germany) under approval number H0032-09 for in vivo PDX passages and A0452-08 for preclinical sensitivity experiments.

### 2.3. Tumor Histology and Immunohistochemistry

For tumor histology, FFPE blocks were sectioned (4–8 μm) and paraffin was removed. After cutting snap-frozen tissues (5 µm), they were fixed in 96% ethanol for 5 min. A standard hematoxylin eosin protocol was applied to specimens for histopathological evaluation. Tissue-Tek embedded tumor fragments were cut into 5 µm sections and fixed in 4% formaldehyde for 1 min. After hydrogen peroxide treatment and blocking with 20% goat serum, the primary antibodies anti-α-SMA (1:200, #ab75005, Covalab, Bron, France) and anti-collagen I (1:250, #ab21286, Abcam, Waltham, MA, USA) were incubated overnight at 4 °C. After incubation of the secondary antibody (anti-rabbit-HRP, 1:200, #IR111-035-003, Jackson Immuno Research, West Grove, PA, USA, 1:400, 30 min, RT), the DAB chromogen (EnVision-mouse HRP, Dako, Santa Clara, CA, USA) was added for 2 min and the counterstaining was performed with hematoxylin (3 min, RT). Proof of IHC staining specificity including the use of negative controls without primary antibody incubation was obtained routinely. Representative pictures from IHC staining were taken using the Axioskop 40 (100-fold magnification) and AxioVision 4.5 (both Zeiss, Jena, Germany). For quantification of α-SMA and collagen I expression, five representative images of three sections of all treatment groups of each PDX model were taken and analyzed using the software ImageJ 1.45 s [38].

### 2.4. In Vivo Chemosensitivity Testing of Pancreas Carcinoma PDX Models

Groups of five female NMRI:nu/nu mice with s.c. PDX were randomized to receive either the solvent as control or one of the respective chemotherapeutic drugs (Gemcitabine at 100 mg/kg i.p., Erlotinib at 5 mg/kg p.o., Abraxane at 50 mg/kg i.v., 5-FU at 80 mg/kg i.p., Oxaliplatin at 2.4 mg/kg i.p.; all given once weekly). Treatment was started at an advanced tumor size of approximately 0.2 cm^3^. For evaluation of therapeutic efficacy, tumor growth of treated and of control mice was measured in two dimensions with a caliper. Tumor volumes (TVs) were determined as described above. Tumors were routinely passaged at TV = 1 cm^3^. The ratio of the mean TV values of the treated group (T) and the control group (C) was expressed as the T/C value in a percentage. T/C ≤ 10% was defined as a strong response, T/C ≤ 25% as a moderate response, T/C ≤ 50% as a minor response and T/C > 50% as resistant. Drug doses were previously evaluated to avoid detrimental side effects in treated animals.

### 2.5. In Vivo Ultrasound-Based Tumor Measurement of Orthotopic PDAC

For tumor volume measurement with ultrasonography, the Vevo^®^ 2100 High Resolution System (FUJIFILM Visualsonics, Tokyo, Japan) was used for B-mode acquisition. Anesthetized animals were placed into the induction chamber and pre-warmed ultrasound gel was applied to the lateral abdominal area. Subsequently, an ultrasound transducer (MS-550S) was used in a 40 MHz range to visualize the pancreas and the PDAC. The tumor was imaged using the software VevoR 2100. For the tumor quantification, an outline was drawn around the tumor for measuring the tumor size in each slice and was then 3D-reconstructed. The tumor volume was quantified using the software VevoLAB 2.2.0.

### 2.6. Total RNA Isolation and cDNA Synthesis

Total RNA from PDX tumor tissue samples was isolated by using the RNeasy Mini Kit (Qiagen, Hilden, Germany) following the manufacturer’s instructions. The RNA was eluted in 50 µL of RNAse-free H_2_O and used for a further analysis. For the reverse-transcriptase reaction, the Reverse-Transcriptase-Kit (Applied Biosystems, Schwerte, Germany) was used according to manufacturer’s instructions, generating a master mix of 1 × RT-buffer (500 μM dNTPs, 5.5 mM MgCl_2_, 2.5 μM random hexamers, 0.4 U/μL of RNase inhibitor, 1.25 U/μL of MultiScribe-Reverse-Transkriptase). Then, 200 ng of RNA was diluted in 10 µL of the RT buffer and the RT-PCR reaction was performed at 25 °C, 10 min; 48 °C, 30 min; and 95 °C, 5 min.

### 2.7. Quantitative Real-Time PCR (qPCR) for Gene Expression Analysis

In total, 200 ng of cDNA, TaqMan^®^ (Schwerte, Germany) Fast Master Mix and a Gene Expression Assay kit were combined in a total volume of 20 µL according to manufacturer’s instructions. The real-time PCR was carried out on a StepOnePlus™ System and was conducted at 95 °C for 10 min, followed by 40 cycles of 95 °C for 15 s and 60 °C for 1 min. Primer IDs—GAPDH, mouse: NM_008084.2, SPARC: Mm00486332_m1, FAP: Mm01329177_m1 (Applied Biosystems). The amplification plots were analyzed using StepOne™ Software Version 2.3. The threshold cycle (CT) of the gene of interest was normalized to the CT of GAPDH and the resulting ΔCT values were used for comparison of expression between samples.

### 2.8. Molecular Characterization of PDX Models by RNA Sequencing

#### 2.8.1. Total RNA Isolation and Sequencing

Total RNA was isolated as described above, disrupting PDX tissue with a TissueLyser (Qiagen) and performing on-column treatment with DNase for removal of genomic DNA (DNase set, Qiagen). For preparation of RNAseq libraries, the Illumina TrueSeq Stranded mRNA Library Prep Kit was used, following a 100 bp PE-sequencing run on an Illumina NovaSeq device with a depth of 80–100 × 10^6^ reads (40–50 Mio cluster) (Illumina, Cambridge, UK). Next-generation sequencing (RNAseq) as well as raw data processing of one untreated PDX tumor tissue sample of each established PDAC model were performed by ATLAS Biolabs GmbH (Berlin, Germany).

#### 2.8.2. Data Processing and Mutational Analysis

The tool Xenome v1.0.1 [39] was used for classifying xenograft-derived sequence reads (human/mouse read splitting), referencing human genome hg38 as the graft reference as well as mouse genome mm10 as the host reference. Variant calling and annotation of mapped reads were conducted with GATK v4.0.2.1 [40] and the Ensemble Variant Effect Predictor (VEP), release 94 [41]. The subsequent mutational analysis was performed for a set of 70 PDAC-relevant genes resulting from the respective data sets in the TCGA (The Cancer Genome Atlas) [42]. In order to identify putative somatic alterations related to the tumor disease, quality-proven variant calls were filtered based on population allele frequencies from gnomAD [43]. Only variants that either were not included in the gnomAD database or have a gnomAD allele frequency below 0.05 were considered.

#### 2.8.3. Data Processing and Gene Expression Analysis

Quality of transcript reads was validated with FastQC v0.11.8 [44]. STAR aligner v2.6.1a [45] was used to map reads against the Homo sapiens’ reference hg38 and quality of mapping was validated with QualiMap v2.2.1 [46]. Human-specific reads were extracted with bamcmp v2.2 [47] and gene counts were calculated with RSEM v1.3.3 [48]. Gene names were used according to the annotations approved of the HUGO Gene Nomenclature Committee (HGNC) [49], resulting in an RNAseq raw count data set of 19160 HGNC-annotated genes for each model. Further data processing and analyses were performed in R v4.2.2 using packages provided by Bioconductor 3.16. After removing batch effects using R package SVA [50], raw counts were normalized to transcripts per million (TPM). The packages gplots [51], RColorBrewer [52] and factoextra [53] were used to visualize the expression data as heatmaps and perform hierarchical clustering (complete linkage, Euclidean distance) and principal component analyses. Scoring for selected signaling pathways or biological processes was performed using single-sample gene set enrichment analyses (ssGSEAs) [54], based on gene sets of the molecular signatures database (MsigDB) and the drug signatures database (DsigDB) [55,56]. GSEA [57] was used to test for enriched gene sets between the groups of responding (strong and moderate) and resistant PDX models.

#### 2.8.4. Human Leukocyte Antigen (HLA) Typing

The individual HLA profiles of 41 PDAC PDX models comprising HLA class I and II alleles in a 4-digit resolution were determined with the computational tool seq2HLA [58] based on RNAseq data. Equally probable allele alternatives were included in further analyses. Allele frequencies (AFs) were calculated as the ratio of the number of occurrences of the corresponding allele and the total number of alleles of the respective HLA locus. For comparative HLA profile analyses, AFs of 8862 healthy German stem cell donors (GSCDs) provided by the Allele Frequency Net Database were examined [59].

### 2.9. Statistical Analyses

Statistical analyses and figure generation were performed in R v4.2.2, using packages provided by Bioconductor 3.16, and GraphPad Prism v10.0.2 (GraphPad Software, Boston, MA, USA). Details of the analyses and packages used are described in the respective sections. *p*-Values below 0.05 were considered statistically significant.

## 3. Results

### 3.1. Patient Tumor Characteristics and Histology of Pancreas Carcinoma PDX Models

About 120 surgical tumor samples from different medical centers were used to stably establish 45 PDAC PDX models. The PDAC PDX cohort originates from 19 female and 26 male patients, representing almost even distribution regarding gender. During establishment, we observed engraftment rates that ranged from 23% to 60%, highly dependent on fast and non-delayed processing of the specimen for transplantation in the recipient mice and a low stroma content. A correlation between take rate and grading or staging of the tumor was not observed. The average time to initial engraftment in NSG mice was 76 ± 32 days. Of the 45 PDAC PDXs, 42 (93%) represented primary PDACs and 3 models were established from liver metastases (Table 1).

**Figure 1:**
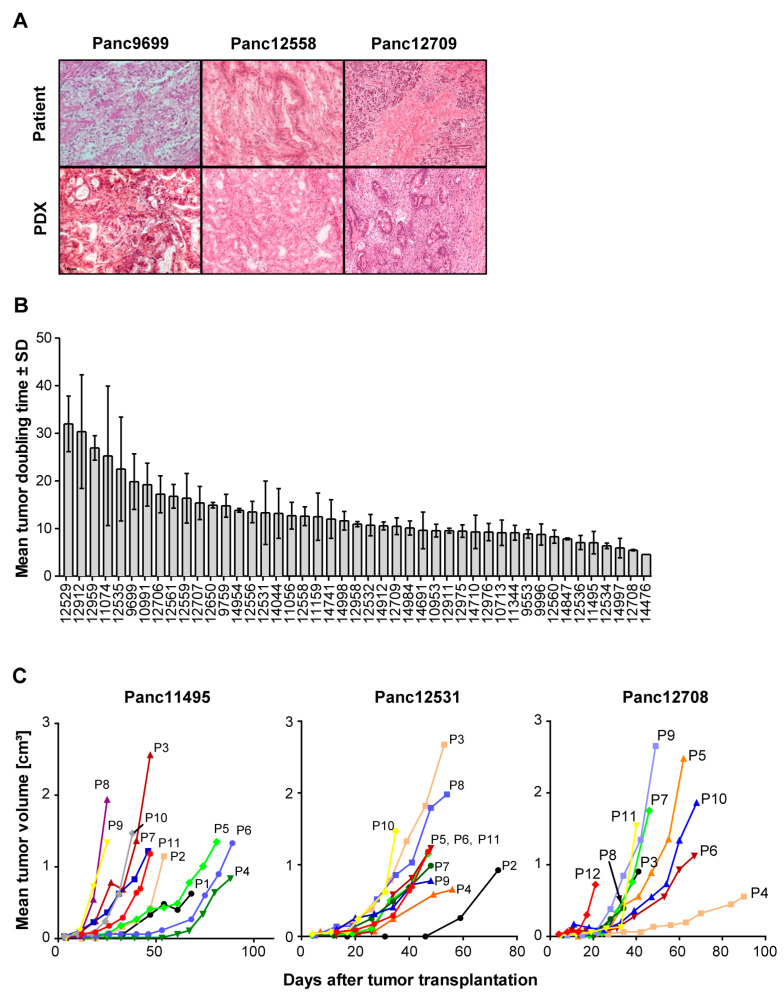
Histological features and growth characteristics of the PDAC PDX models. (**A**) Comparison of the histology of the primary patient tumor tissues (upper panel) and of the corresponding PDX tissues (lower panel) in H&E-stained tissue slices. Magnification: 40×. (**B**) Mean tumor doubling times (TDTs) of all 45 PDAC PDX models showing high range of the TDT for these models ranging from 5 days to up to >30 days. Values represent means of 2 to 6 measurements with respective SD values. *n* = 5 mice. (**C**) Alterations in TDTs shown for three representative PDAC PDX models during consecutive passages (from passage P1 up to passage P12), indicating accelerated PDX growth with increasing passage number on mice. *n* = 3 to 5 mice.

The histology of the PDX models closely resembles the original histology of the patient tumors (Figure 1A). Interestingly, the proportion of stroma in these tumors is comparable with the proportion of stroma in the patient tumors. In conclusion, PDAC-specific histological architecture is preserved in the PDX tumors, with mixed composition of human tumor areas and surrounding mouse stroma.

### 3.2. Growth Characteristics of PDX Models

Since for use of in vivo models their growth characteristics are of importance, we also determined the tumor doubling times (TDTs) of all PDX models over up to 11 consecutive in vivo passages (Figure 1B,C). Overall, we observed changes in TDT for all PDAC PDX models, which changed from slow TDT of 32 to 5 days in early passages (P0 to P4) to predominantly accelerated TDT of 17 to 4 days in the late passages (P5 to P10) of the respective PDX models. This is an indication of tumor adaptation to the mouse host in the in vivo environment, leading to improved growth of the tumor models.

### 3.3. Chemosensitivity of PDX Models

**Figure 2:**
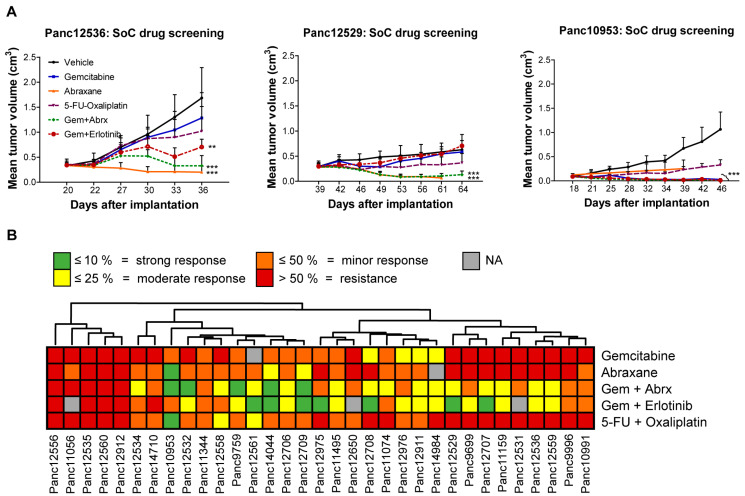
Sensitivity testing of PDAC PDX models toward SoC drugs in subcutaneous setting. (**A**) Mean tumor volume of s.c. PDX models during and after treatment with various SoC drugs (gemcitabine, abraxane, 5-FU, oxaliplatin) and one targeted drug (erlotinib), as well as drug combinations (gemcitabine/erlotinib; gemcitabine/abraxane; 5-FU/oxaliplatin). Tumor growth was monitored regularly from day 20 to 54 and the tumor volume was determined; *n* = 5 mice per group. Three representative PDX models (Panc12536, Panc12529 and Panc10953) with different levels of sensitivity toward drug treatment are shown, reflected by changes of mean tumor volumes. (**B**) Unsupervised clustering of PDAC PDX models according to categorized T/C response rates of the individual treatment regimens is showing sensitivity and resistance characteristics for all PDX models. ** *p* < 0.01, *** *p* < 0.001.

For characterization of the PDAC PDX models, their responsiveness toward standard of care (SoC) drugs in monotherapy or combination therapy in clinically relevant and optimized schedules and dosages was tested. The response criterion was met if the treated-to-control tumor volume (T/C) was ≤ 50%. PDAC models Panc12529, Panc125356 and Panc10953 illustrate the individual response to the applied treatment regimen over time (Figure 2A). Panc10953 showed complete response to gemcitabine monotherapy and in combination with erlotinib or abraxane (T/C ≤ 10%), while Panc12536 and Panc12529 showed only partial remission to the combination of gemcitabine and abraxane (T/C ≤ 25%) in comparison to gemcitabine alone. Combination therapy of 5-FU and oxaliplatin showed progressive disease for all three tested PDAC models (Figure S1).

Throughout the cohort of 33 tested PDAC PDX models, 45% of models responded to gemcitabine and 83% to the abraxane monotherapy. The combination of both drugs revealed synergistic effects in a small cohort of 5 PDXs (Figure 2B).

**Figure 3:**
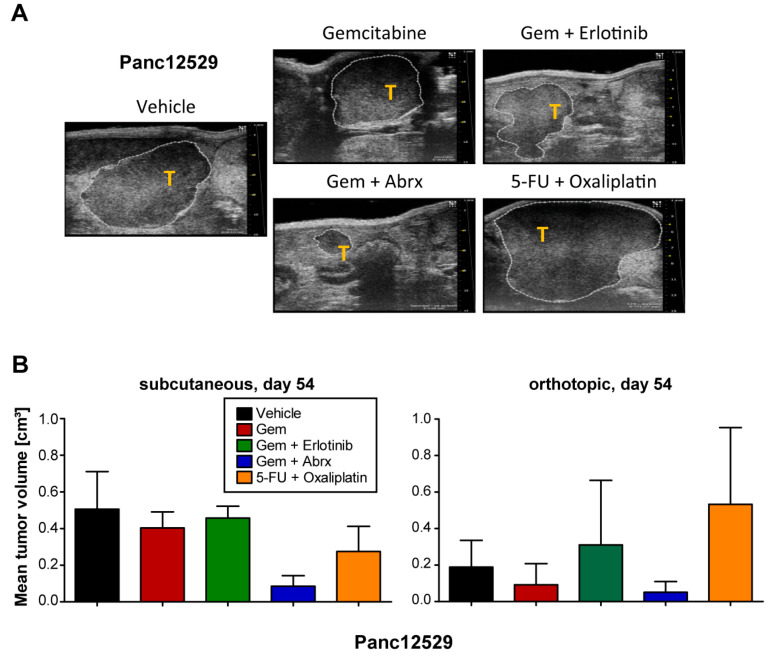
Comparison of treatment responses in subcutaneous and orthotopic settings. (**A**) Tissue samples of the PDAC PDX model Panc12529 were transplanted orthotopically, receiving the above-mentioned treatment regimen. Measurement of tumor volumes in the orthotopic setting by using ultrasound measurements of tumors (T) within the pancreas of mice and respective calculation of tumor volumes with software VevoLAB 2.2.0. (**B**) The resulting tumor volumes in both settings are given as mean tumor volumes + SD.

To extend our sensitivity analyses, we also compared drug sensitivity to the SoC drugs and combinations thereof in the PDAC PDX model Panc12529, after s.c. or orthotopic transplantation (Figure 3A,B). Treatment response of the orthotopic model was determined by calculation of tumor volumes from ultrasound measurements (Figure 3A). The s.c. model showed no response to gemcitabine and its combination with erlotinib, while we observed reduced tumor volumes upon gemcitabine treatment in the orthotopic model. The combination of gemcitabine and abraxane resulted in partial remission in both transplantation routes. Combination treatment of 5-FU and oxaliplatin showed a stronger response in the s.c. model, compared to the orthotopic model. Overall, the transplantation route very likely influenced the response of the PDX tumor to SoC treatment, but the resulting responses followed the same trend.

### 3.4. Impact of Transplantation Route and Stroma on Chemosensitivity

**Figure 4:**
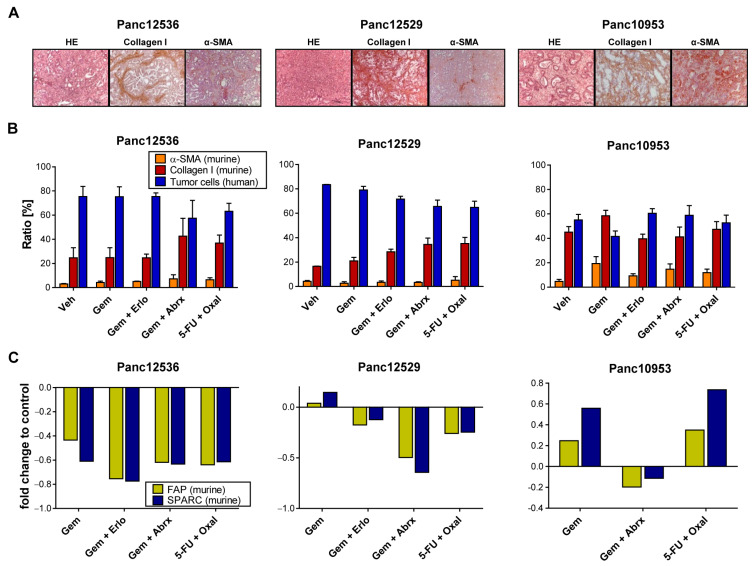
Stromal factors and their impact on PDAC chemotherapy. (**A**) HE staining of tumor tissues illustrates individual histomorphological features of the PDAC PDX models Panc12536, Panc12529 and Panc10953; magnification 40-fold. Protein expression grades of the stroma marker α-SMA and collagen I were determined with IHC. (**B**) Semi-quantitative evaluation of α-SMA and collagen I protein expression was performed at 5 different sight fields of stained tumor sections of each treatment group. The ratio of positive stained areas was related to the total area and given as %-ratio + SD. (**C**) Fold change in mRNA expression of the prognostic biomarkers FAP and SPARC, analyzed with qRT-PCR in treated tumors of s.c. PDX models.

Therapy response of PDAC tumors is strongly influenced by its stromal content. Since we observed similar histomorphological characteristics of human PDAC tumor tissue and its corresponding PDX model, we were interested in if and how the stromal composition is altered during various treatment regimens. IHC analyses of murine collagen I and α-SMA show strong protein expression of the cancer-associated fibroblast (CAF) markers in the PDX tumors, indicating full replacement of the human stroma with mouse stroma (Figure 4A).

We followed the proportion of human PDAC cells and murine CAFs with IHC of collagen I and α-SMA in tumor sections of Panc12529, Panc125356 and Panc10953 after treatment and quantified stained stroma cells (Figure 4B). In general, we observed no alteration in the ratio of CAFs in all tested PDAC models after treatment, compared to the control. The ratio of α-SMA-expressing cells was below 10% in all treatment groups, with no differences between s.c. and orthotopic transplantation (Figure S2). Panc10953 is characterized by a higher ratio of stroma components in comparison to Panc12536 or Panc12529. The ratio of collagen I positivity in all treatment groups was higher than 40%.

Further, we determined murine mRNA expression levels of the prognostic biomarkers SPARC and FAP in s.c. and all three orthotopic PDX models after drug treatment (Figure 4C and Figure S3). Due to lack of specific antibodies against murine FAP and SPARC, TaqMan qRT-PCR was chosen for the determination of both markers. mRNA levels of SPARC and FAP were rather low, with only minor differences comparing s.c. and orthotopic PDAC models after treatment with SoC drugs. Hence, the expression of SPARC and FAP had no effect on tumor growth and treatment response in these PDX models.

### 3.5. Mutational Analysis

**Figure 5:**
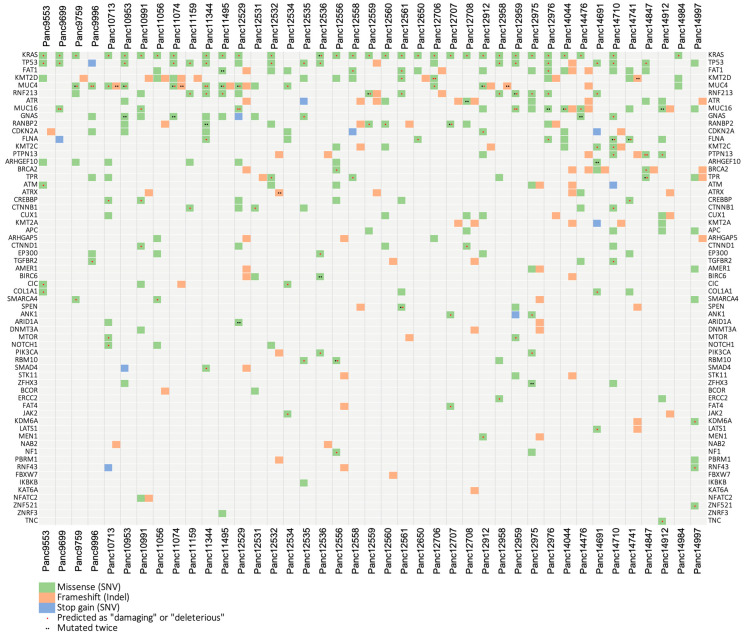
Mutational landscape of the pancreas carcinoma PDX cohort. Using variant calls from human RNA sequencing data, 70 selected genes that are frequently mutated in the TCGA PDAC cohorts were analyzed for putative somatic mutations in 41 PDAC PDX models. We found putative somatic mutations in 62 different genes and in all 41 examined PDX models. Sequence variations were filtered based on population allele frequencies from the gnomAD database. Only variants that either were not included in gnomAD or have a gnomAD allele frequency below 0.05 were considered.

Performing RNASeq with a high sequencing depth of 80–100 million reads allowed a sensitive mutational analysis of the transcriptome of 41 of our PDAC PDX models. In the analysis, we focused on the top 100 frequency-sorted mutated genes found in relevant TCGA-GDC data sets including 2.337 pancreatic cases. We found putative somatic mutations in 62 different genes and in all 41 examined PDX models (Figure 5, Table S1). All models cover more than one gene aberration, with Panc12529—originated from a liver metastasis—showing by far the highest and Panc14984 showing the lowest mutation rate. The most mutated genes from this list found in our PDX cohort are (from high to low) *KRAS*, *TP53*, *FAT1*, *KMT2D*, *MUC4*, *RNF213*, *ATR*, *MUC16*, *GNAS*, *RANBP2* and *CDKN2A*. This is similar to the frequency pattern that can be found in the relevant case cohorts hosted by the TCGA-GDC database. By far the most frequently mutated genes in the pancreatic tumor cohorts are *KRAS* with 75% of affected patients and *TP53* with 65% followed by *CDKN2A* and *SMAD4* with 20%, respectively. This situation is well reflected by our pancreatic PDX model set. Almost all models (80%) exhibit a deleterious or protein damaging mutation in *KRAS* and 50% in *TP53*. An exception is *SMAD4*, which is apparently less mutated in our xenograft cohort, compared to the TCGA data.

Since the gene set enrichment analysis of RNAseq data revealed a quite differentiating picture of the expression of DNA damage repair pathways in our pancreatic cancer models, we additionally selected 39 genes playing a major role in six DDR pathways for a mutational analysis [60,61] (Figure S6, Table S2).

### 3.6. Expression Analysis and Pathway Activities

We molecularly characterized 41 PDAC models with RNAseq to eventually correlate expression patterns or cancer-relevant signaling pathways in the human tumor cells with the responses to the various treatment regimens. Individual expression patterns of the PDAC PDX cohort were scored for the activity of cancer hallmark pathways (MsigDB). An unsupervised clustering of differences in the hallmark activities is shown in Figure S4. Similarly, we estimated alterations in the copy number of larger chromosomal regions of the cohort, resulting in distinct patterns of amplifications or deletions within the PDAC PDX models (Figure S5).

**Figure 6:**
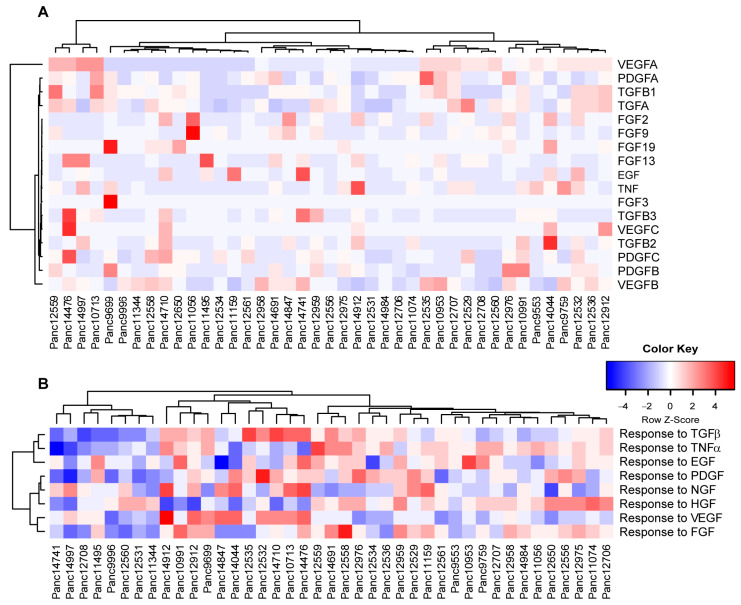
PDAC PDX models reflect the heterogeneity in tumor/stroma interactions. (**A**) Unsupervised clustering of selected growth factor expression in human tumor cells. Factors that expressed below 10 TPM in all models were excluded from the analysis. (**B**) PDX transcriptomes were scored for the enrichment of human gene sets related to the cellular response of the indicated growth factors (GOBP, MsigDB) and hierarchically clustered.

As mentioned above, the tumor stroma in patient PDAC tumors has a significant impact on the potential response during anti-cancer therapies. Taking into account that the tumor cells themselves can shape the murine stroma into a tumor supporting environment, we determined the expression pattern for various human growth factors to stimulate stromal response (Figure 6A). Unsupervised clustering of growth factor expression by human tumor cells showed vascular endothelial growth factors (VEGFs) A, B and C as the strongest differentiators within the cohort, potentially stimulating stroma-driven angiogenesis. In turn, we compared potential responses in the human tumor cells to (growth) factors released by the murine tumor stroma (Figure 6B), indicating clusters of models responsive or rather inert to stromal signaling.

**Figure 7:**
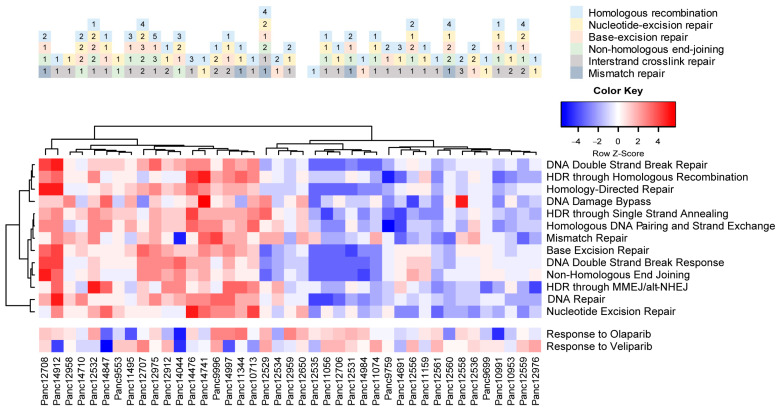
Predicted DNA damage repair activity and potential response to PARPi therapy in the PDAC PDX models. Occurring mutations in DNA damage repair factors impact the intrinsic or induced repair activity of the cells (upper panel). Pastel colors indicate the DDR pathways; numbers indicate the mutated factor within each pathway. Gene set enrichment analysis of DDR pathways results in differentiating models with high intrinsic DNA damage repair activity, as well as potential sensitivity toward PARP inhibitors alone or in combination with DNA-damage-inducing agents.

As indicated by the mutational characteristics of the PDAC PDX cohort, we found a variety of mutations (SNV, frame shift) in proteins associated with DNA damage repair pathways (Figure S6). We also analyzed the potential activity or deficiency of individual repair pathways, like homologous recombination (HR), base and nucleotide excision repair (BER and NER, respectively), non-homologous end joining (NHEJ) and mismatch repair (MMR). We observed enriched gene sets of DNA repair pathways in almost half of the cohort, indicating higher actual DNA damage repair activity in those models (Figure 7). A similar analysis of the transcriptomes also allowed for scoring each model for enriched gene sets associated with cellular response to olaparib and veliparib, indicating potential response of the PDX models to a treatment with PARP inhibitors.

### 3.7. Human Leukocyte Antigen (HLA) Typing

**Figure 8:**
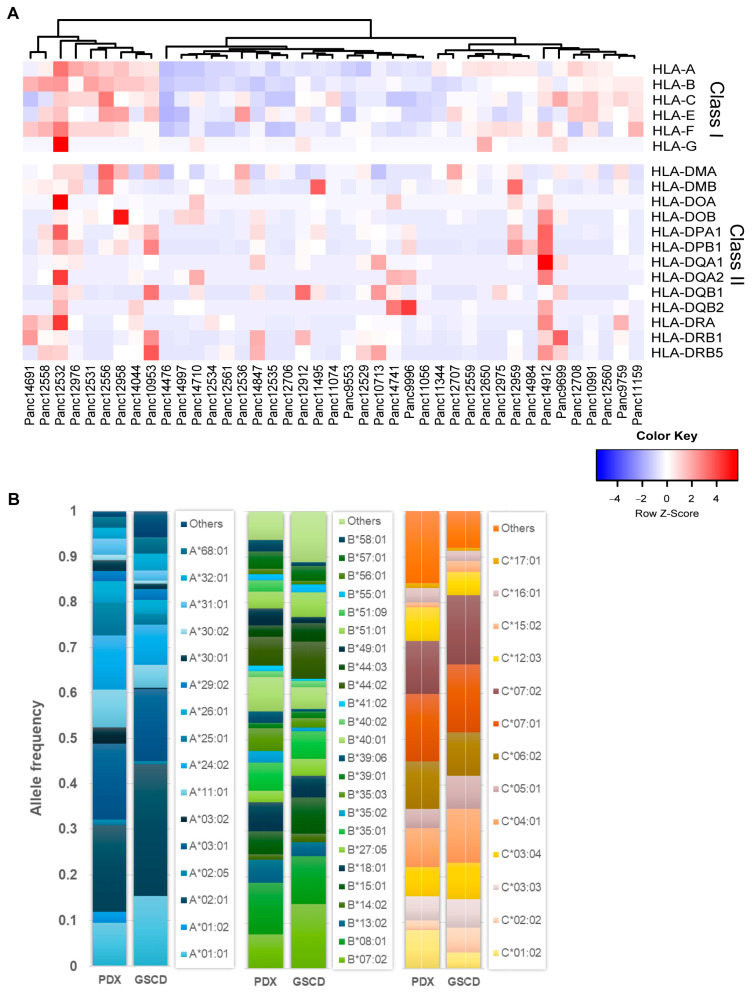
HLA profile of 41 PDAC PDX models. (**A**) Unsupervised clustering of PDX models ac-cording to their gene expression (TPM) of HLA class I and II loci. (**B**) Comparison of HLA-A, -B, -C (class I) allele frequencies (AFs) of PDX models and a representative population of 8862 healthy German stem cell donors (GSCDs). The PDX HLA profiles showed comparable AF to the GSCD HLA profiles.

The HLA system plays a crucial role in the antigen-specific immune response. The highly polymorphic HLA antigens determine the histocompatibility of donor and recipient tissue and are associated as diagnostic and prognostic markers with a variety of diseases. Here, individual HLA profiles of 41 PDAC PDXs were generated comprising HLA class I and II loci. The heatmap shown in Figure 8A represents the relative expression of HLA genes in the PDX models. An unsupervised clustering of the expression data revealed three main clusters reflecting PDX models with similar expression patterns, for example, a high (left cluster), low (middle cluster) and moderate (right cluster) level of class I gene expression.

In an exemplary fashion, Figure 8B shows the detected HLA alleles in a 4-digit resolution for class I loci HLA-A, -B and -C. To estimate the representativeness of the generated PDX HLA profile panel, it was compared with a healthy population of 8862 German stem cell donors (GSCDs). In the PDAC PDX cohort, 17 different HLA-A alleles were detected; in the GSCD population, 61 were detected. In total, 16 out of 17 PDX alleles match those of the GSCD population and account for about 94% allele frequency (AF) of GSCD. For HLA-B, 24 out of the detected 28 were also found in the 101 types of GSCD, corresponding to an AF of 89%. For HLA-C, 13 out of 14 alleles were also detected in the 47 GSCD alleles accounting for 92% AF. In summary, these results showed comparable frequencies of alleles determined in both PDX and GSCD HLA profiles.

**Figure 9:**
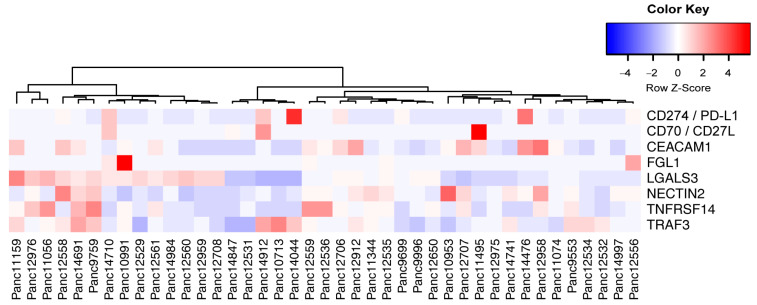
Expression relation of immune checkpoint ligands for potential immune therapy. Unsupervised clustering of selected immune checkpoint ligand expression in PDAC PDX tumor cells indicate the possibility to evaluate targeted immune therapies in preclinical trials. Factors that expressed below 10 TPM in all models were excluded from the analysis.

HLA profile matching of PDX models and human immune cells allows the evaluation of novel drugs that aim in enhancing immune responses in PDAC treatment. To select the most suited model for preclinical studies, we also analyzed the expression of novel immune checkpoint ligands in the PDAC PDX cohort (Figure 9) [62].

## 4. Discussion

Pancreas ductal adenocarcinoma (PDAC) remains a complex disease associated with the worst outcomes for patients. Although great efforts are made to improve therapies for PDAC, the mortality of this disease is still increasing and might represent the second cancer-related cause of mortality by 2030 [63]. PDX models of PDAC are a key platform that contributes to better understanding of the molecular biology, molecular basis of therapy response and testing of novel therapy concepts. Numerous studies have demonstrated the value of such models for more personalized PDAC therapies and demonstrated their effectiveness in predicting efficacy of standard-of-care as well as novel anticancer drugs [64,65,66,67,68].

In this study, we aimed at stable establishment of PDAC PDX models and their thorough characterization regarding morphology, in vivo growth, chemosensitivity and molecular features, including mutational and expression analyses with RNAseq, and HLA profiling. This resulted in a biologically and molecularly well-characterized PDX panel, which adds to other already established and published PDAC PDX panels with a similar comprehensive data set [34,69,70].

The comparison of histologic features of patient tumors and the corresponding s.c. and orthotopic PDX tumors in our models has shown that tissue morphologies and cellular structures resemble the adenocarcinoma phenotype and remain the same, even though human stroma was replaced by murine stroma. The maintenance of histological architecture and patient-characteristic heterogeneity even over many passages of PDAC PDX was also described for other PDAC PDX panels [69,71]. Thus, the murine stroma rather adequately complements the tumor morphology for the PDX models.

The engraftment rate of our PDX cohort varied from 23% to 60% and is similar to results of Pham et al. (52%), Pergolini et al. (43%) and Garrido-Laguna et al. (61%) [37] [72,73]. Others report that the take rate strongly depends on the recipient mouse strain, as shown for 35 primary patient tumors, which generated a significantly higher take rate in NSG (97.3%) versus NOD/SCID mice (57.1%) [64]. One key issue for PDX models is the similarity of the original tumor with its PDX derivative, and numerous studies addressed this issue. In this context, Chen et al. generated 30 xenograft models from 67 patients (take rate = 44.8%) and this cohort recapitulated the pathology and genetic characteristics of the primary tumors [74]. Genetic consistency between PDX models and patient tumors regarding copy number alterations and genomic instability was confirmed by Genta et al. [75].

We also characterized our PDAC PDX panel regarding responsiveness toward SoC drug treatments in s.c. settings and we observed a response rate of 40% toward gemcitabine, as the most important therapeutic drug for PDAC. This reflects the observations in other PDX studies well. In a study of Garrido-Laguna et al., 22% of the PDX models responded to gemcitabine treatment and induced resistance in another 22% [73]. In a study of Hou et al., 8 out of 17 (47%) PDX models responded to gemcitabine and the authors were able to establish a prognostic panel of five genes for gemcitabine response [76]. A response rate of 47% of PDX models to gemcitabine was also found by Hoare et al. [77]. Of note, the PDX models were most representative with a wide phenotype scope, compared to xenograft-derived pancreatic organoids and xenograft-derived primary cell cultures of the same patient.

Compared to s.c. model setup, orthotopic models are considered more clinically relevant, as they better mimic the human pancreatic cancer microenvironment and favor the development of metastases more closely [78]. Further, it is assumed that these models provide a better prediction of treatment success in patients than s.c. xenograft models [79]. However, s.c. transplantation allows continuous monitoring and measurement of tumor growth. Considering the advantages and disadvantages of s.c. and orthotopic settings, we compared selected PDX models regarding their chemosensitivity in both settings but observed no significant differences. This is an important indication that the s.c. transplantation of PDAC is well suited to appropriately predict responsiveness and resistance of drug treatments in preclinical studies.

PDAC is characterized by a rather dense stroma, which consist of an extracellular matrix, cancer-associated fibroblasts (CAFs) and immune cells [80,81]. Prognostic biomarkers such as SPARC, FAP, collagen I and α-SMA were chosen to characterize murine CAFs in our PDX models. Both biomarkers, SPARC and FAP, are associated with tumor progression and metastasis and predict a poor outcome in patients with PDAC [82]. And we detected low mRNA expression rates of SPARC and FAP in all tested PDX models. Several studies have shown that a depletion in FAP-positive CAFs restricted tumor growth by enhancing antitumor immunity, which occurs through multiple mechanisms, including loss of immunosuppressive effects of stromal cells or enhancement of tumor survival [83,84]. Chlenski et al. identified rare myofibroblasts in subcutaneous xenografts with SPARC expression, whereas a large number of α-SMA-positive fibroblasts was apparent in tumors with low SPARC expression [85]. However, the ratio of α-SMA-positive cells remained unchanged in the untreated PDX models Panc12536 and Panc12529, either transplanted s.c. or orthotopically. The transplantation route also had no influence on the mRNA expression of SPARC and FAP in all three PDX models (Figure S3). Our analyses showed that the tumor-surrounding mass was not reduced in response to therapeutic intervention. Although the tumor burden was diminished under SoC treatment, the mRNA expression levels of SPARC and FAP were unaffected in corresponding samples of the treatment groups compared to vehicle-treated controls. Furthermore, the transplantation route did not influence the expression of α-SMA, SPARC and FAP, but we found a higher ratio of collagen I in orthotopic PDX models and a decrease in corresponding tumor cells.

The modulation of tumor/stroma interaction, especially in PC, is under both preclinical and clinical evaluation to decipher the involved signaling pathways and response patterns, and to find novel therapeutic targets that interfere with critical processes therein. By releasing a variety of growth factors (FGFs, PDGF, TGFs, etc.), PDAC cells are able to shape their microenvironment toward tumor support, e.g., by activating pancreatic stellate cells (PSCs), which can induce the proliferation of tumor cells, but also may differentiate into CAFs [86]. This crosstalk of human PDAC cells with murine stroma can, at least in part, explain the higher occurrence of CAFs in orthotopic PDAC PDX models. Looking at VEGF signaling, with VEGFA as the strongest discriminating growth factor expression within the cohort, the clinical use of bevacizumab alone or in combination with SoC therapy did not meet the high expectations, similar to other anti-angiogenic treatment strategies [87,88]. Nevertheless, modulating the vascularization of the tumor can be a novel option to deliver therapeutic agents more efficiently throughout advanced tumors, compared to passive and ECM-restricted diffusion [88].

By analyzing the mutational landscape of our PDAC PDX cohort, we observed several models with mutations in DNA damage repair pathways, especially in factors regulating homologue recombination of double-strand breaks, like BRCA1/2, ATR and ATM. Since those cells rely on alternative repair mechanisms of intrinsic or therapy-induced DNA damages, and as most of these mechanisms involve the activity of PARP, the use of PARP inhibitors can improve the disease outcome when it can be combined with DNA-damaging SoC therapies [61]. Olaparib monotherapy is already approved by the FDA for the treatment of the BRCA1/2 mutant, homologous recombination repair-deficient pancreatic cancer, and Parsels et al. [89] could show that olaparib-mediated DNA damage is amplified by ATR inhibition and correlates with radiosensitization. The combination of olaparib and the ATR inhibitor AZD6738 inhibited tumor growth significantly in mice together with radiation therapy. Lloyd et al. [90] also showed a therapeutic activity of olaparib and AZD6738 in xenograft and PDX mouse models with complete ATM loss, and recommended clinical investigation of AZD6738 in combination with olaparib. In addition, at least one established model in our cohort can be considered mismatch-repair-deficient, as we determined an unusually high mutation rate and low scores for the respective gene set. The occurrence of MMR deficiency is rather low in PDAC tumors, compared to colorectal cancer [91], and their high mutational load also leads to the generation of tumor neo-antigens. In most cases, the growing tumor has evolved strategies to evade the immune surveillance, e.g., overexpressing immune checkpoint ligands like PD-L1, which renders them susceptible to immune therapies like checkpoint inhibitors [89,90].

Preclinical in vivo studies of immune efficacy on PDX tumor growth are often hamstrung by unspecific allograft reactivity of applied immune cells. One way to overcome this hurdle is to find HLA-compatible tumor/immune models, in which a specific anti-tumor response of the immune cells can be modulated by immune enhancers or checkpoint inhibitors. HLA typing of 41 PDAC PDXs and the comparison with the HLA profiles of 8862 GSCDs indicated that our panel can reflect the HLA allele distributions of a large, representative cohort. Among other factors, the obtained variations in the determined alleles and frequencies could be attributed to differences between a healthy population and patients with PDAC. Actually, differences in the expression of certain HLA genes can serve as prognostic markers. For example, higher expression of HLA-A, -B, -C, -E and -G on PDAC cells has been associated as a prognostic marker for shorter survival of the respective cells [92]. In immune–oncology research, comprehensive HLA profile portfolios, similar to ours, provide important information for the generation of humanized mouse models. HLA profile matching of mutually compatible PDX models and human immune cell populations enables personalized, preclinical studies to promote the development of new immune–therapeutic strategies. In turn, comprehensively characterized PDX cohorts are invaluable tools for translational research, as they simulate the patient diversity in clinics.

## 5. Conclusions

Our PDX panel reflects the heterogeneous pheno- and genotype of PC and can contribute to identifying biomarkers for tumor progression and therapy response at an experimental stage. When working with PDX, everyone should keep the limits of these models in mind [66,93]: maintenance of human tumor tissues in mice selects the fast adapting population of cancer cells, altering the clonal heterogeneity of the PDX tumor, while the influential human tumor stroma is replaced by murine analogues and the tumor-intrinsic immune cells are lost. However, no other preclinical model provides this high heterogeneity of tumor cells and similarity of morphologic and genomic features with patient material [29,37]. The combination of molecular profiles, information on the ECM, as well as immune-related data provide a broader basis for the development of novel drugs in a preclinical setup.

## Figures and Tables

**Table 1:** Key clinical data of patients from whom PDAC PDX models were established.

PDX ID	Gender	Age	Histology	TNM/Grading	Kras Mutation	Classification
9553	F	67	PDAC	pT3 N1/G3	G12D	Primary
9699	M	70	PDAC	pT3 pN1 L1 V1 Pn1/G3	G12V	Primary
9759	M	61	PDAC	pT3 pN1 L1 V0 Pn1 R1/G3	G12R	Primary
9996	F	60	PDAC	pT3 pN1/G3	wildtype	Primary
10713	M	64	PDAC	pT4 N1/G3	G12D	Primary
10953	F	61	PDAC	pT3 N1/G2	G12R	Primary
10991	F	72	PDAC	pT3 N1/G3	G12V	Primary
11056	M	77	PDAC	pT3 N1/G2	G12D	Primary
11074	F	76	PDAC	pT3 N0/G3	Q61H	Primary
11159	M	76	PDAC	pT3 N1/G3	wildtype	Primary
11344	M	69	PDAC	pT3 N1/G3	G12V	Primary
11495	F	51	PDAC	pT3 N1/G3	G12V	Primary
12529	F	53	PDAC	pT4 pN2 M1, Stage IV/G1	G12V	Liver met
12531	M	54	PDAC	pT4 pN1 M1, Stage IV	wildtype	Liver met
12532	F	72	PDAC	pT4 pN1 M1, Stage IV	G12D	Liver met
12534	F	71	PDAC	pT3 N1 M1/G3	wildtype	Primary
12535	M	46	PDAC	pT3 N0 M1	wildtype	Primary
12536	F	69	PDAC	pT3 pN1 M0, Stage IV	Q61H, T58I	Primary
12556	M	67	PDAC	pT3 N1 M0, Stage IIB	G12V	Primary
12558	M	56	PDAC	pT3 N1 M0, Stage IIB	G12D	Primary
12559	M	60	PDAC	pT3 N1 M0, Stage IIB	G12D	Primary
12560	F	82	PDAC	pT3 N0 M0, Stage IIA	G12R	Primary
12561	F	80	PDAC	pT3 N1 M0, Stage IIB	G12R	Primary
12650	M	48	IPMN *	pT3 N0 M0, Stage IIA	G12D	Primary
12706	F	82	IPMN *	pT3 N0 M0, Stage IIA	G12V	Primary
12707	M	60	PDAC	pT3 N1 M0, Stage IIB	G12V	Primary
12708	F	56	PDAC	pT3 N1 M0, Stage IIB	G12D	Primary
12709	F	51	PDAC	pT3 N1 M0, Stage IIB	G12D	Primary
12911	M	67	PDAC	pT3 N0 M0, Stage IIA	G12V	Primary
12912	M	67	PDAC	pT3 N1 M0, Stage IIB	G12D	Primary
12975	F	75	IPMN *	pT3 N0 M0, Stage IIA	G12R	Primary
12976	F	65	PDAC	pT3 N1 M0, Stage IIB	G12D	Primary
12958	M	83	PDAC	pT3 N1 M0	G12R	Primary
12959	M	44	PDAC	pT3 N1 M0	G12D	Primary
14044	M	65	PDAC	pT3 N0 M1 L1	G12V	Primary
14476	M	65	PDAC	pT3 N1 L0	Q61K	Primary
14741	M	50	PDAC	pT2 N1 L0	wildtype	Primary
14912	M	73	PDAC	pT2 N1 L1	wildtype	Primary
14954	F	63	PDAC	pT2 N0 L0	n/a	Primary
14984	M	50	PDAC	pT3 N1 L0	Q61H	Primary
14997	M	64	PDAC	pT2 N1 L1	wildtype	Primary
14998	M	75	PDAC	pT2 N0 L0	n/a	Primary
14691	M	65	PDAC	pT3 N1 M0/G2	wildtype	Primary
14710	F	68	IPMN *	pT3 N0 M0/G3	G12D	Primary
14847	M	80	PDAC	pT3 N1 M0 L1/G3	wildtype	Primary

* Associated with invasive ductal adenocarcinoma; n/a—not available.

## Data Availability

Data sets generated and/or analyzed during the study are available upon reasonable request.

## Supplementary Materials

The following supporting information can be downloaded at: https://www.mdpi.com/article/10.3390/cancers15245753/s1, Figure S1: Response to SoC treatment of subcutaneous PDX models; Figure S2: Ratio of human tumor cells and murine CAFs stained with a-SMA and collagen I of orthotopic Panc12536 and Panc12529; Figure S3: The fold gene expression of murine SPARC and FAP in subcutaneous and orthotopic PDX models treated with SoC drugs; Figure S4: Enrichment of gene sets associated with general cancer hallmarks; Figure S5: Large-scale genomic copy number alterations of the PDAC PDX cohort; Figure S6: Relevant sequence variations found in DDR-pathway-related genes in the PDAC PDX cohort with RNASeq; Table S1: Relevant sequence variations found in pancreas carcinoma PDX with RNASeq as visualized in Figure 5; Table S2: Relevant sequence variations found in DDR-pathway-related genes in pancreas carcinoma PDX with RNASeq as visualized in Figure S6. References [60,61,94,95] are cited in the supplementary materials.
