# Supplementary material for: Establishment and Thorough Characterization of Xenograft (PDX) Models Derived from Patients with Pancreatic Cancer for Molecular Analyses and Chemosensitivity Testing
Source: Cancers (Basel). 2023 Dec 8;15(24):5753. doi: 10.3390/cancers15245753 (PMC10741928; doi:10.3390/cancers15245753)
Supplement: Supplementary file 1 [file cancers-15-05753-s001.zip › Behrens_Cancers2023_SuppFigures.pdf]

Establishment and Thorough Characterization of Xenograft (PDX) Models Derived from Patients with Pancreatic Cancer for Molecular Analyses and Chemosensitivity Testing

Diana Behrens, Ulrike Pfohl, et al, Cancers (MDPI) 2023

Supplementary Figures

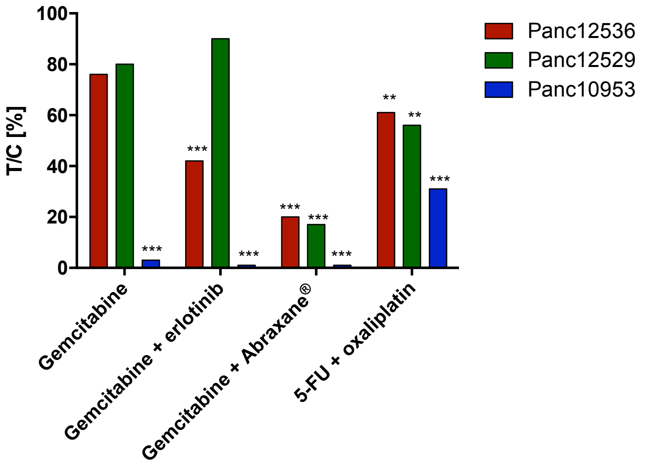

Figure S1: Response to SoC treatment of subcutaneous PDX models. The percentage of tumor volumes of treated models versus control (T/C) was determined by dividing the mean tumor volume in the treatment group through the mean volume in the control group at the final day of treatment for Panc12536 (red), Panc12529 (green) and Panc10953 (blue). $**p < 0.01$, $***p < 0.001$ (ANOVA)

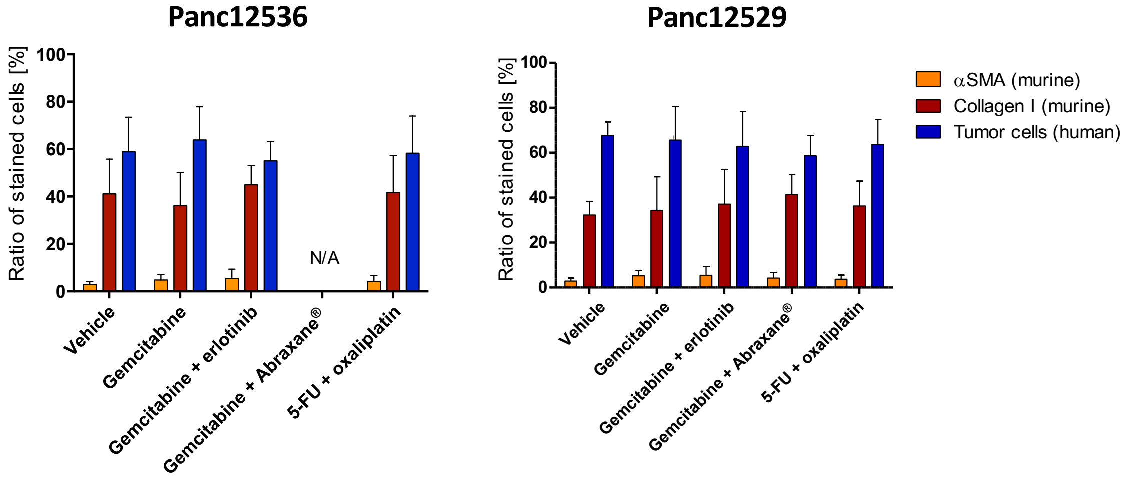

Figure S2: Ratio of human tumor cells and murine CAFs stained with α-SMA and collagen I of orthotopic Panc12536 and Panc12529. Paraffin-embedded tissue sections were stained for murine α-SMA to detect activated fibroblasts. Cryosections were stained for murine collagen I to detect fibroblasts in general. Immunohistochemical staining was quantified by using Image J. Five pictures of the same section and in total three replicates of each s.c and orthotopic tumor (four animals per treatment group) were analyzed.

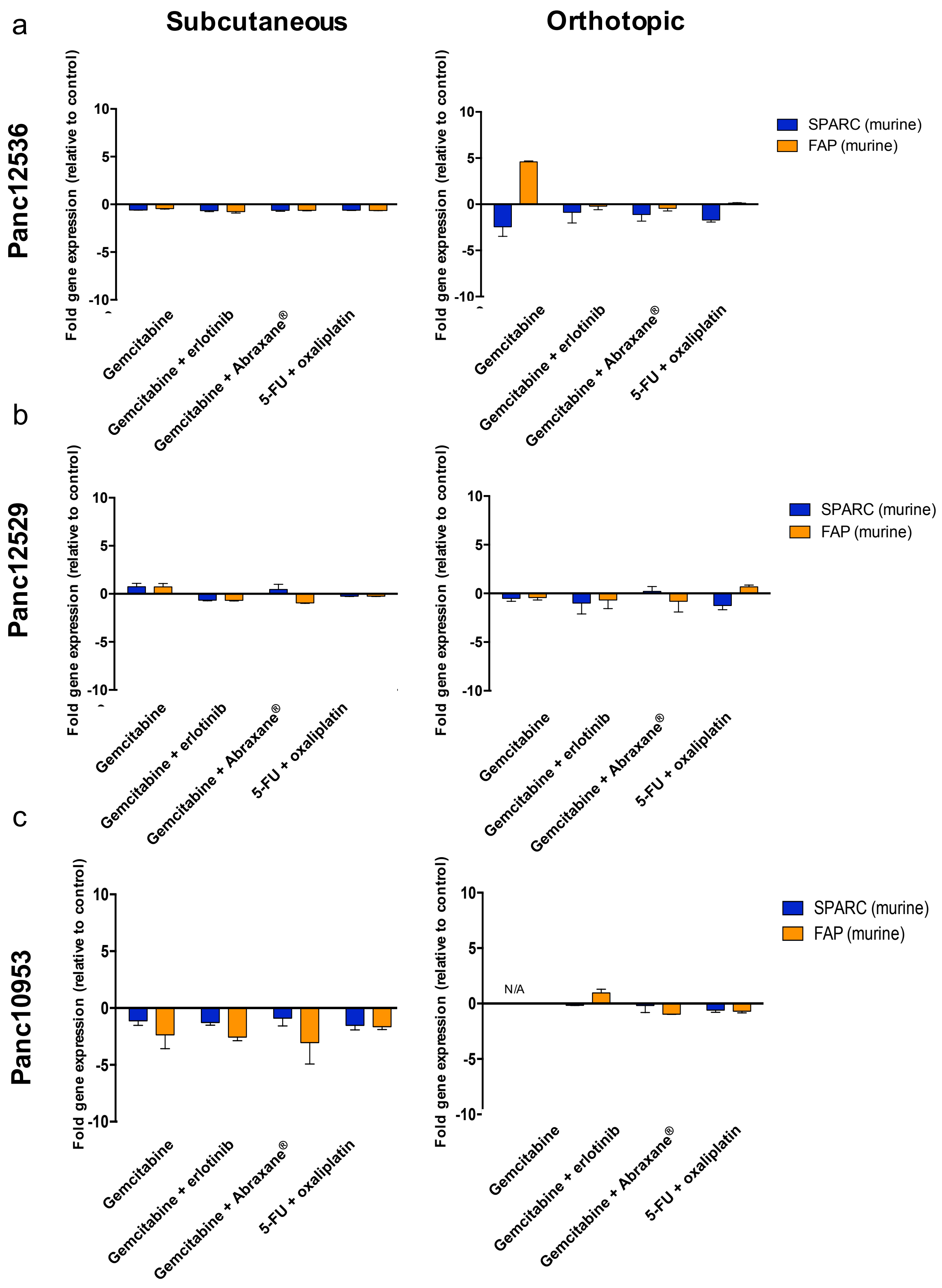

Figure S3: The fold gene expression of murine SPARC and FAP in subcutaneous and orthotopic PDX models treated with SoC drugs. Quantitative real-time PCR (TaqMan™) was used to analyze the gene expression of murine stroma markers SPARC and FAP of Panc12536 (a), Panc12529 (b) and Panc10953 (c) subcutaneously transplanted (left row) and orthotopically transplanted (right row) by using the $2^{-\Delta\Delta CT}$ method. Animals were treated with gemcitabine, gemcitabine plus erlotinib, gemcitabine plus Abraxane® and 5-FU plus oxaliplatin. Data from the gemcitabine group of orthotopic Panc10953 were not available (N/A) as tumors were too small..

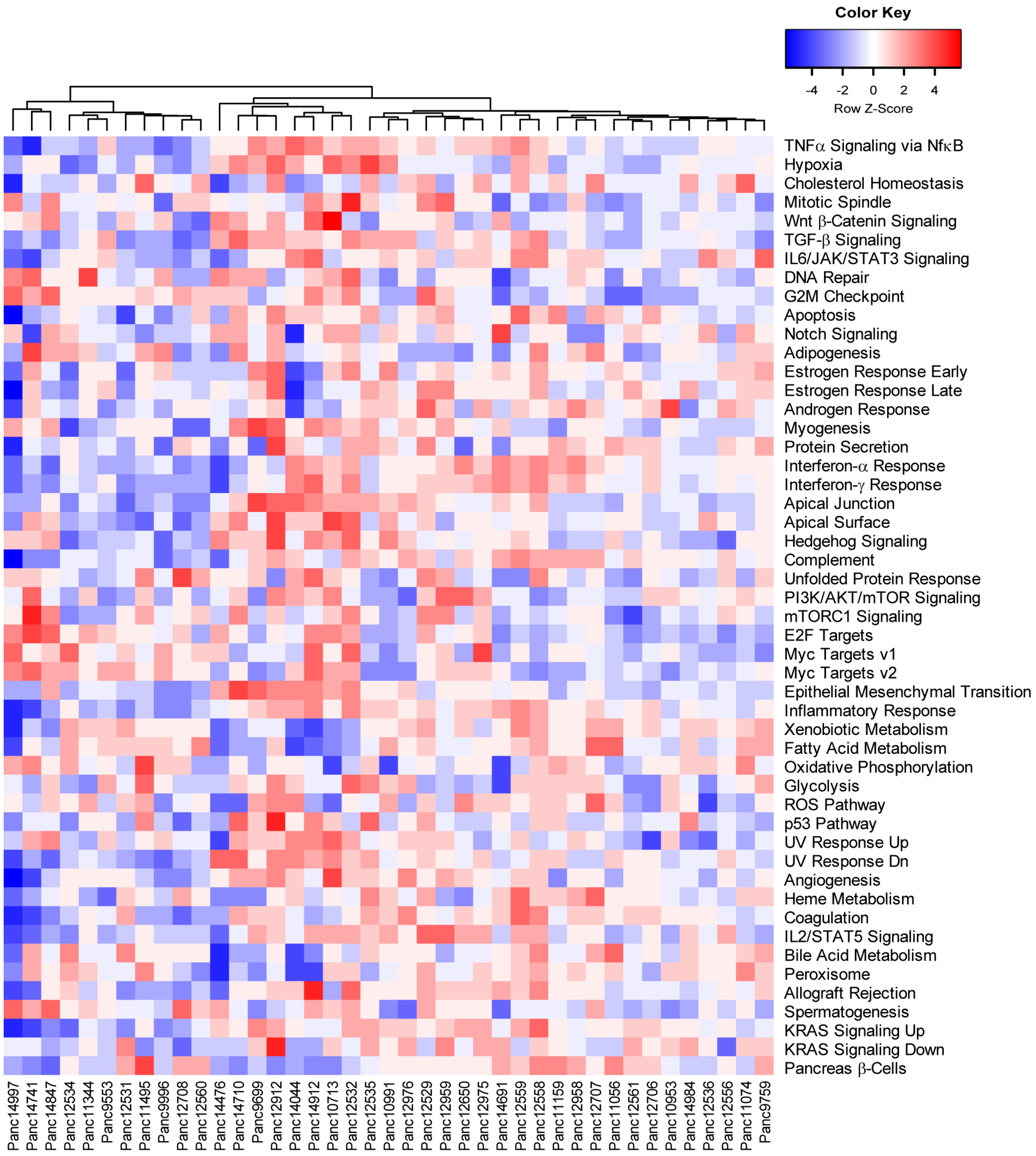

Figure S4: Enrichment of gene sets associated with general cancer hallmarks. TPM-transformed gene counts of 41 PDAC PDX models were analyzed for enriched gene sets of cancer hallmarks (MsigDB) and resulting scores were hierarchically clustered according to their similarity in deviation from the group median (z-score).

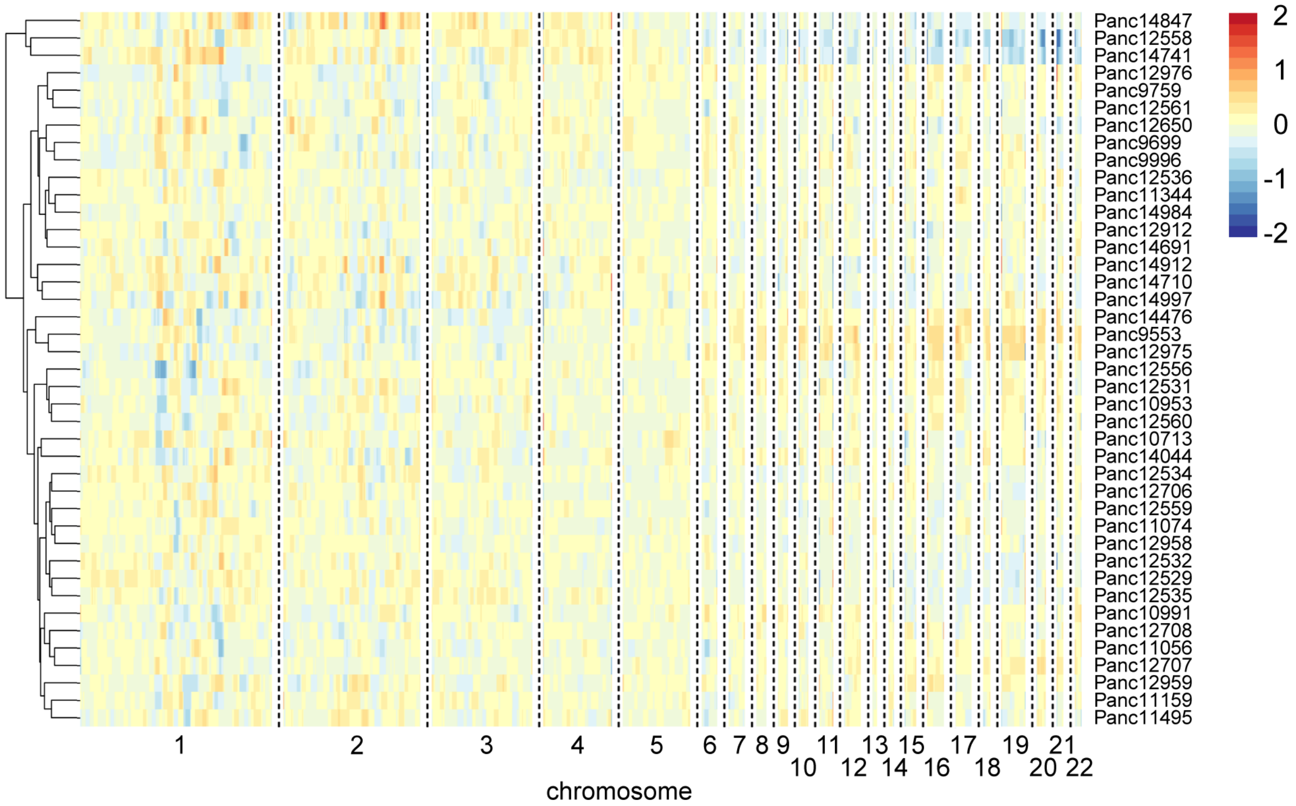

Figure S5: Large scale genomic copy number alterations of the PDAC PDX cohort. Transcriptomic data (raw gene counts, SNVs) for each model was used to estimate copy number alterations in larger genomic regions. CNV data on chromosome arm level were calculated with RNAseqCNV (Barinka, et al., **Leukemia** 36, *2022*) [94] with subsequent estimation of B-allele frequencies via CaSpER (Harmanci, et al., **Nat Comm** 11, *2020*) [95]. Resulting estimates in CNVs of larger chromosomal regions were clustered according to their similarity.

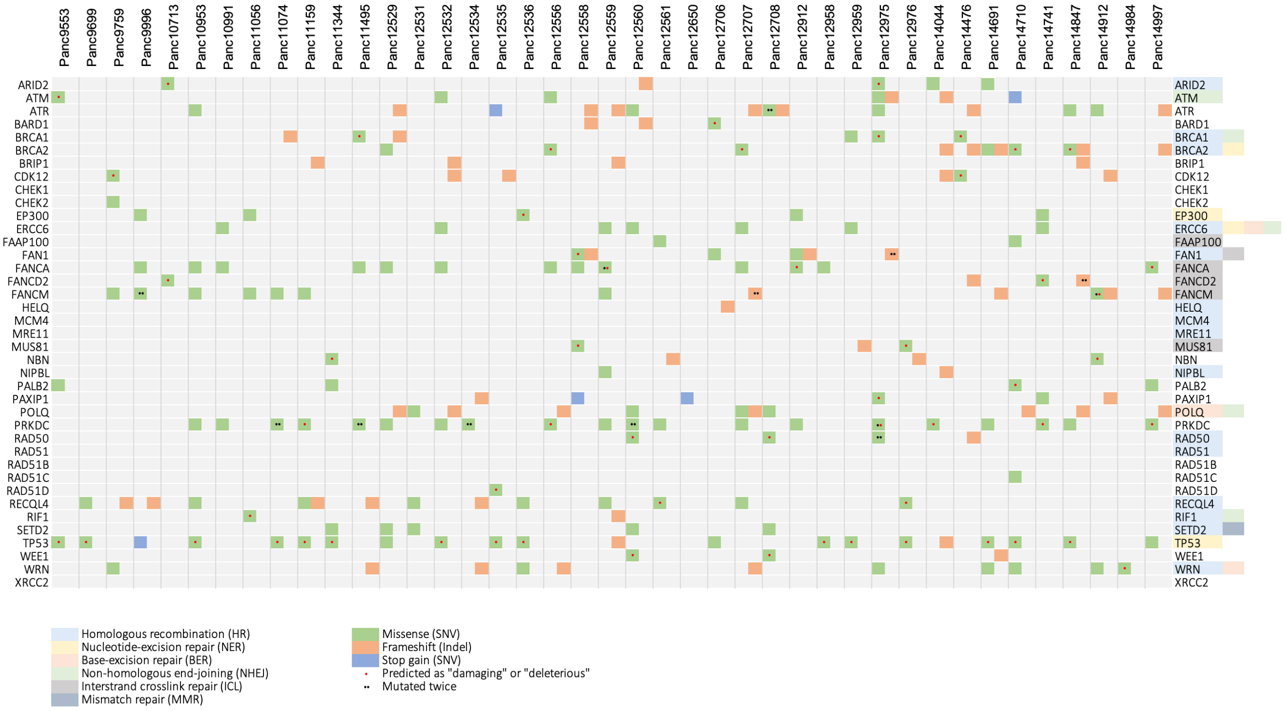

Figure S6: Relevant sequence variations found in DDR pathway related genes in the PDAC PDX cohort by RNASeq.

Since gene set enrichment analysis of RNAseq data revealed a quite differentiating picture of the expression of DNA damage repair (DDR) pathways in our pancreatic cancer models we additionally selected 39 genes playing a major role in 6 DDR pathways for mutational analysis [60, 61] (see corresponding table S2).
